# The apparent prevalence, the true prevalence

**DOI:** 10.11613/BM.2022.020101

**Published:** 2022-06-15

**Authors:** Farrokh Habibzadeh, Parham Habibzadeh, Mahboobeh Yadollahie

**Affiliations:** 1 Global Virus Network, Middle East Region, Shiraz, Iran; 2 Research Center for Health Sciences, Institute of Health, Shiraz University of Medical Sciences, Shiraz, Iran; 3 Freelance Researcher, Shiraz, Iran

**Keywords:** seroepidemiologic studies, prevalence, sensitivity, specificity, diagnostic tests

## Abstract

Serologic tests are important for conducting seroepidemiologic and prevalence studies. However, the tests used are typically imperfect and produce false-positive and false-negative results. This is why the seropositive rate (apparent prevalence) does not typically reflect the true prevalence of the disease or condition of interest. Herein, we discuss the way the true prevalence could be derived from the apparent prevalence and test sensitivity and specificity. A computer simulation based on the Monte-Carlo algorithm was also used to further examine a situation where the measured test sensitivity and specificity are also uncertain. We then complete our review with a real example. The apparent prevalence observed in many prevalence studies published in medical literature is a biased estimation and cannot be interpreted correctly unless we correct the value.

## Introduction

Serologic tests are commonly used in seroepidemiologic and prevalence studies (*1*). The design is typically conducted to understand the current situation of a condition of interest, say a disease. For example, over the past two years, soon after the announcement of the coronavirus disease pandemic, many serologic tests have been developed for diagnosis of the severe acute respiratory syndrome coronavirus 2 (SARS-CoV-2); numerous seroepidemiologic studies have been conducted to determine the prevalence of the disease in various parts of the world. For example, a population-based seroprevalence study revealed a SARS-CoV-2 seroprevalence of 9.7% in the Principality of Andorra (*2*). The results obtained from seroepidemiologic studies are generally used by health care researchers to understand where we do stand by estimating the health burden and the economic impact of a disease, and policy-makers to better identify the priorities and planning (*3*). But, are the values obtained from these studies valid?

At the heart of the design is the method by means of which we identify the condition of interest. We usually use a diagnostic test to detect the condition (*e.g.*, a disease). However, a diagnostic test is usually not perfect; it may give false-positive and false-negative results; not all people with positive tests are diseased, and not all with negative tests are disease-free (*4*). This is why the prevalence derived from these studies, the so-called “apparent prevalence” (*pr*), is not necessarily an unbiased estimation of the true prevalence (*π*), the true proportion of diseased people in the population or the study sample. Herein, we are going to discuss how we can derive an unbiased estimation of *π* from the obtained *pr* and the test sensitivity (*Se*) and specificity (*Sp*). We also used a computer simulation program to better investigate the situation.

## Prevalence

The *pr* (the apparent prevalence) is defined as the portion of tested people with a positive test (*T* ^+^) (*5*). Therefore:



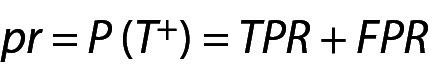



where *TPR* and *FPR* are true-positive and false-positive rates, respectively. Substituting the *TPR* and *FPR*, we have (*4*):



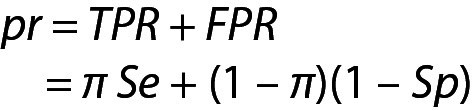



Solving the above equation for *π* (the true prevalence), yields:



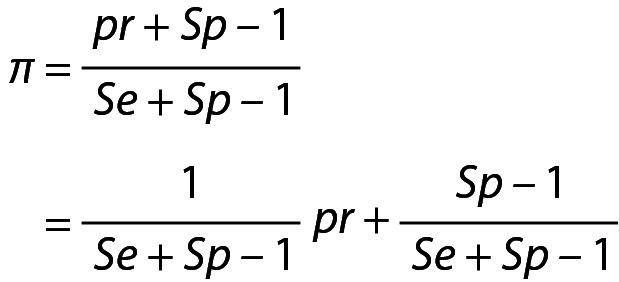



**Figure 1:**
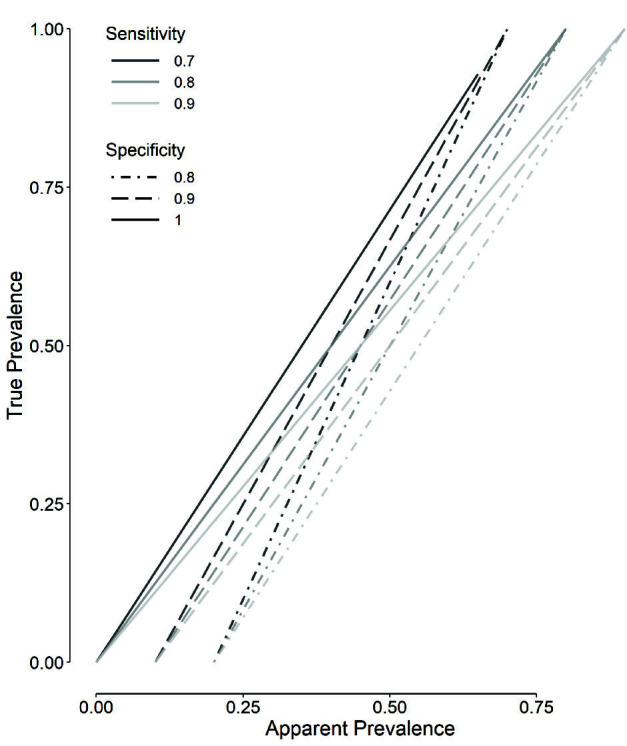
The linear relationship (Eq. 3) between the true and the apparent prevalence for a number of combinations of the test sensitivities and specificities.

This shows that the true prevalence (*π*) and the apparent prevalence (*pr*) are linearly related (Figure 1). If we take into account the uncertainty existing in the measured estimates of *pr*, *Se*, and *Sp*, Eq. 3 becomes:



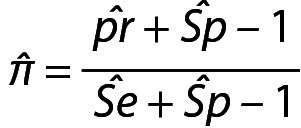



where *x* (any variable with a hat, *e.g.*, or *pr*) represents an estimation for *x* (*e.g.*, *π* or *pr*). Assuming that *pr* and the test *Se* and *Sp* are independent, employing basic calculus and using a first-order Taylor series expansion, we have (*6*, *7*):



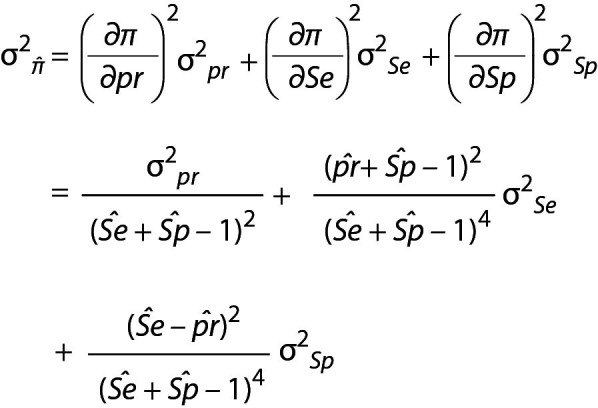



where *σ*^2^_x_ represents the variance of *x*. Based on the results, we can calculate the 95% confidence interval (CI) of the true prevalence (*8*-*10*). To portray the effect of variations in estimates of *pr*, *Se*, and *Sp* on the *π*, we conducted a Monte-Carlo simulation program.

## Computer simulation

We assumed that the *Se* and *Sp* of a diagnostic test were measured in a hypothetical validity study on 225 individuals: 75 in whom the disease was confirmed and 150 without the disease (Table 1). The results gave a *Se* of 93% (95% Cl 88% to 99%, *σ*^2^_Se_ = 8.3 x10^-4^) and a *Sp* of 90% (85% to 95%, *σ*^2^_Se_ = 6.0 x10^-4^).

**Table 1:** Results of the hypothetical test validity study

		**Disease**		**Total**
		**Present**	**Absent**	
**Test**	**Positive**	70 (*TP*)	15 (*FP*)	85
	**Negative**	5 (*FN*)	135 (*TN*)	140
**Total**		75	150	225
TP - True positive. FP - False positive. FN - False negative. TN - True negative. N = TP + FP + FN + TN = 225. Se = TP/(TP + FN) = 0.93. Sp = TN/(TN + FP) = 0.90. TPR = TP/N = 0.31. FPR = FP/N = 0.07. FNR = FN/N = 0.02. Apparent prevalence = TPR + FPR = 0.31 + 0.07 = 0.38. True prevalence = TPR + FNR = 0.31 + 0.02 = 0.33. Using Eq. 4. it can be calculated: True prevalence = (Apparent prevalence + Sp - 1)/(Se + Sp - 1) = (0.38 + 0.90 -1)/(0.93 + 0.90 - 1) = 0.33.				

To further investigate the situation, we used a Monte-Carlo simulation (Table 2, Supplementary material). We assumed an arbitrarily chosen population size of 1,000,000 people and assumed that 200,000 of whom had a disease - *i.e.*, a population true prevalence of 0.20. We randomly selected a sample of 300 individuals from the population. Each person in the study sample was then tested with a diagnostic test with *Se* and *Sp* values randomly selected from the above-mentioned distributions (supposed to be Gaussian with a mean of 93% and variance of 8.3 x10^-4^ for the *Se*, and a mean of 90% and variance of 6.0 x10^-4^ for the *Sp*) (Table 2). The *π_s_*, the proportion of individuals in the sample with the disease (true prevalence of the disease in the sample); the *pr_s_*, the proportion of people in the sample with a positive test (apparent prevalence of the disease in the sample); and the calculated true prevalence (in the sample), *π_c_*, derived from Eq. 4 and 5, were then estimated for each sample. The above steps were repeated for an arbitrarily chosen 200,000 samples. The frequency distributions of *π_s_*, *pr_s_*, and *π_c_* were then plotted and compared. Linear regression analysis (no intercept model) was used to determine the relationship between the *π_s_* and *π_c_*.

**Table 2:** Pseudocode of the simulation program

Begin
Determine the *Se* and *Sp* distribution from a validation study
Construct a *Population* of 1,000,000 people; 200,000 of whom are diseased
*Loop* for 200,000 times
Choose a random sample (N = 300) from the *Population*
Choose a *Se* and *Sp* from the *Se* and *Sp* distributions
Calculate *π_s_* = *P*(*D*^+^), *pr_s_* = *P*(*T* ^+^), and *π_c_* (Eq. 4 and 5)
*EndLoop*
Draw the frequency distributions of *π_s_*, *pr_s_*, and *π_c_*
End
*D*^+^ - Having the disease. *T* ^+^ - Test-positive. *P*(*x*) - Probability of *x. Se* - sensitivity. *Sp* - specificity. *π_s_* - true prevalence. *pr_s_* - apparent prevalence. *π_c_* - calculated true prevalence. For more details, see the R codes in the Supplementary materials.

## Simulation results and discussion

**Figure 2:**
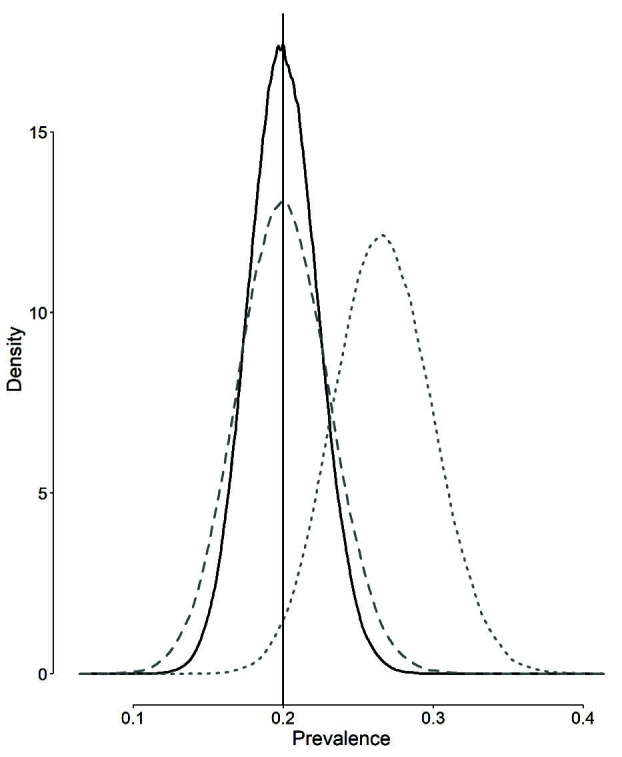
The frequency distribution of the true prevalence (*π_s_*, solid curve), seroprevalence (*pr_s_*, dotted gray curve), and the calculated true prevalence (*π_c_*, dashed gray curve) derived in 200,000 rounds of simulation on 300 individual samples. The black vertical line is the population true prevalence (*π*) of 0.2.

**Figure 3:**
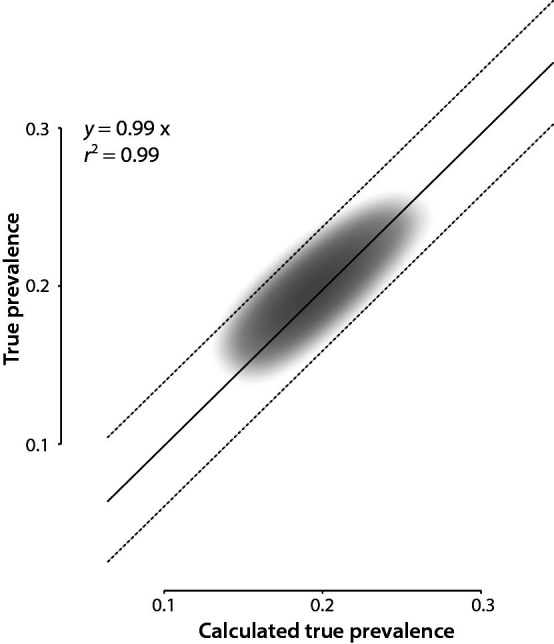
The scatter plot of true prevalence (*π_s_*) against the calculated prevalence (*π_c_*). The solid line is the linear regression line (no intercept model); dashed lines represent the regression 95% confidence interval.

The mean true prevalence (*π_s_*) was 0.20 (95% CI 0.16 to 0.25) – as expected, equal to the population true prevalence (*π*) of 0.20. The mean apparent prevalence (*pr_s_*) was 0.27 (0.20 to 0.33), a biased estimate of the true prevalence (*π_s_*) (Figure 1). The mean calculated true prevalence (*π_c_*), 0.20 (0.14 to 0.26), however, was an unbiased estimation for the true prevalence (*π_s_*) (Figure 2). The slope of the regression line was almost 1; the model could explain almost all of the variance observed in the *π_c_* (Figure 3). The observed variance of the *π_s_* distribution was less than that of the *pr_s_* (Figure 2). The former was attributed to the sampling variation; the second, to the sampling variation and variability in the test *Se* and *Sp* distribution used for each sample. The variance of the *π_c_* distribution (similar to that of the *pr_s_*) was also due to the variations in estimating the *pr_s_* and the test *Se* and *Sp* (Eq. 4). It is important to note that the term “test” in this context should be construed in a general way as any means for classifying individuals, either a laboratory test for checking a biomarker, an imaging procedure examination, or a physical examination to check presence or absence of a sign (*11*, *12*). To elaborate on the topic presented, let us examine the following example.

### Example

In the first round of a population-based seroprevalence study on SARS-CoV-2 serological screening, conducted in the Principality of Andorra, the researchers found that 6816 of 70,389 tested people were seropositive, translating into a seroprevalence, *pr_s_*, of 9.7% (95% CI 9.5% to 9.9%) (*2*). The *Se* and *Sp* of the diagnostic test they used (Livzon rapid test, Zhuhai Livzon Diagnostics Inc, Guangdong, China) were 92% (84% to 96%) and 100% (95% to 100%). The values were derived from a validation study conducted on 48 diseased and 48 disease-free individuals (*2*). Here, the *pr_s_*, of 9.7% does not reflect the correct portion of the population with previous exposure to SARS-CoV-2; there might be several people with false-positive test results due to cross-reacting antibodies, technical issues, *etc.*, some people might have false-negative tests, on the other hand (*13*). The seroprevalence (the apparent prevalence, *pr_s_*) was an unbiased estimation of the true prevalence, only if the *Se* and *Sp* of the test used would have been equal to 100%, the gold standard test.

Based on the provided data, it is possible to calculate the variances of the seroprevalence, and the test *Se* and *Sp*, which are 1.2 x10^-6^, 8.9 x10^-4^, and 1.5 x10^-4^, respectively. Substituting the values in Eq. 4 and 5, the estimated true prevalence (*π_s_*) is 10.5% (95% CI 8.2% to 12.9%), the correct proportion of the population with previous exposure to SARS-CoV-2. Had merely binomial distribution been used for the calculation of the 95% confidence interval (ignoring the uncertainty in the estimated *Se* and *Sp*) instead of Eq. 5, we would have come to a 95% confidence interval of 10.3% to 10.8%, a much narrower interval.

## Conclusion

Depending on the *Se* and *Sp* of the diagnostic test used in a given prevalence study, the results obtained are generally biased estimates of the true prevalence of the condition of interest (*e.g.*, a disease). The derived apparent prevalence values should therefore be corrected. Based on the variances of the seroprevalence, and the test *Se* and *Sp*, it is possible to calculate an unbiased estimation of the true prevalence.

## Data Availability

The R codes are available from the journal website as Supplementary material. Running the codes results in the data file.
